# Allergenic Can f 1 and its human homologue Lcn-1 direct dendritic cells to induce divergent immune responses

**DOI:** 10.1111/jcmm.12616

**Published:** 2015-07-27

**Authors:** Beate Posch, Christian Irsara, Fabian S Gamper, Martin Herrmann, Daniel Bindreither, Dietmar Fuchs, Norbert Reider, Bernhard Redl, Christine Heufler

**Affiliations:** a Department of Dermatology, Medical University InnsbruckInnsbruck, Austria; b Department of Anaesthesiology and Critical Care Medicine, Medical University of InnsbruckInnsbruck, Austria; c Division of Molecular Pathophysiology, Biocenter, Medical University InnsbruckInnsbruck, Austria; d Division of Medical Biochemistry, Biocenter, Medical University InnsbruckInnsbruck, Austria; e Division of Molecular Biology, Biocenter, Medical University InnsbruckInnsbruck, Austria

**Keywords:** Can f 1, human monocyte-derived dendritic cells, immune response, Lcn-1, lipocalin allergens

## Abstract

Why and when the immune system skews to Th2 mediated allergic immune responses is still poorly characterized. With two homologous lipocalins, the major respiratory dog allergen Can f 1 and the human endogenous, non-allergenic Lipocalin-1, we investigated their impact on human monocyte-derived dendritic cells (DC). The two lipocalins had differential effects on DC according to their allergenic potential. Compared to Lipocalin-1, Can f 1 persistently induced lower levels of the Th1 skewing maturation marker expression, tryptophan breakdown and interleukin (IL)-12 production in DC. As a consequence, T cells stimulated by DC treated with Can f 1 produced more of the Th2 signature cytokine IL-13 and lower levels of the Th1 signature cytokine interferon-γ than T cells stimulated by Lipocalin-1 treated DC. These data were partially verified by a second pair of homologous lipocalins, the cat allergen Fel d 4 and its putative human homologue major urinary protein. Our data indicate that the crosstalk of DC with lipocalins alone has the potential to direct the type of immune response to these particular antigens. A global gene expression analysis further supported these results and indicated significant differences in intracellular trafficking, sorting and antigen presentation pathways when comparing Can f 1 and Lipocalin-1 stimulated DC. With this study we contribute to a better understanding of the induction phase of a Th2 immune response.

## Introduction

Dendritic cells (DC) act at the interface of innate and adaptive immunity orchestrating signals from both to induce the most suitable immune response to a given antigen. In the T-cell area of the lymph node DCs find the rare antigen specific naïve Th lymphocytes which can differentiate into at least four subpopulations of effector T cells that have distinct functions and express hallmark cytokines 1, depending mainly on the cytokines present during the activation process. Interleukin (IL)-12 promotes the differentiation of Th1 cells, characterized by the expression of interferon (IFN)γ. Interleukin-6 and transforming growth factor (TGF)-β induce the differentiation of Th17 cells which produce IL-17A, IL-17F and IL-22. Interleukin-4 is required for the expansion of Th2 cells producing IL-4, IL-5 and IL-13. Interleukin-10 and TGF-β are the cytokines necessary for the differentiation of regulatory T cells which upon differentiation express IL-10 and TGF-β 2–4. This differentiation of the T cell populations, directed by the crosstalk of the antigen with the antigen presenting DCs and several signals from the innate immune system is the main bias towards the kind of the developing immune response 1.

Investigations in recent years have shown that most of the major as well as minor mammalian allergens causing respiratory sensitization and one major food allergen belong to the lipocalin family 5,6. Lipocalins have several different biological functions: They can act as transport proteins for small, principally hydrophobic molecules 7,8. However, they also function as modulators of cell growth and metabolism or regulators of immune response 7,9. Although the physiological function of many lipocalins became obvious during the last years, the mechanism of their allergenicity is still enigmatic. It was suggested that one factor explaining the allergenicity of lipocalins could be a molecular mimicry between endogenous and exogenous lipocalins 10. In fact, all members of the lipocalin family are characterized by a common tertiary structure 11. However, the amino acid sequence similarity between endogenous and exogenous lipocalins might be low, typically below 35% 7. On the other hand, high sequence similarities also exist. For example, the human tear lipocalin Lcn-1 and the homologous major dog allergen Can f 1 have 61% sequence identity 12,13. In this study these two highly homologous proteins are used to compare the immune responses induced by an allergenic and an endogenous lipocalin in DCs.

Dendritic cells, together with epithelial cells, have been identified as master regulators of allergic airway inflammation by providing specific stimuli for the differentiation of the Th2 cell subset 14. In humans, lung DC subsets have been defined and found to exert specific functions that can be associated with distinct expression of endocytic receptors, cell-surface molecules and anatomical location within the lung 15,16. To sample antigen in the airway, epithelium DCs extend processes between epithelial cells, allowing allergens to be taken up by DCs 17. It is feasible to assume that lipocalins are available to lung DCs for take-up. Of the lung DC subsets characterized in mice, monocyte-derived DCs and CD11b^+^ DCs have been shown to be sufficient to prime for Th2 cell immunity 18. Human monocyte-derived DCs were used in this study to analyse the response of DCs to two homologous lipocalins without and with allergenic potential, the human Lcn-1 and the major dog allergen Can f 1, respectively.

## Materials and methods

### Preparation of recombinant lipocalins

For bacterial expression codon optimized genes encoding the mature Lcn-1 (aa 19-176), the mature Can f 1 (aa 19-174) and the mature Fel d 4 (amino acids 16-185) were synthesized with attached SphI and Bam HI restriction sites (for Can f 1 and Lcn-1 and Fel d 4) and the mature major urinary protein (MUP, gene bank accession number NG_016729; amino acids 16–181) with attached restriction sites NcoI and XhoI. The synthetic genes were cloned into pQE-70 (Qiagen Inc, Hilden, Germany) for Can f 1, Lcn-1 and Fel d 4 or pET21d for MUP. The recombinant proteins were produced with a C-terminal His tag in *E.coli*. Purification was performed by a combination of Ni-affinity chromatography and size-exclusion chromatography. A single band at 18.5 kD was obtained in a coomassie blue stained PAGE (Fig. S1). Contaminating LPS was removed by Detoxi-Endotoxin Removing Gel (Thermo Fisher Scientific, Rockford, IL, USA). Protein preparations were determined by the Limulus assay to be near to endotoxin free (<0.01 EU/μg or less than 2 pg/ml in our assay).

Recombinant Lcn-1 produced in human cells was purchased from Sino Biological Inc., Beijing, China.

### Fluorescent dye labelling of lipocalins

Protein labelling and purification kits with Alexa 488 and Alexa 594 were purchased from Molecular Probes (Invitrogen, Eugene, OR, USA) and used according to the manufacturer’s protocol.

### Cell preparations

Generation of monocyte-derived DCs: Human DCs were prepared from peripheral blood monocytes essentially as described 19,20. Anonymous human blood components were obtained from the local blood bank (Central Institute for Blood Transfusion and Immunology, Medical University Innsbruck, Innsbruck, Austria) according to the guidelines of the local blood bank approved by the independent ethics committee of the Medical University Innsbruck and the tenets of the Helsinki Protocol.

On day 6, cells were cultured for 2 additional days with or without 10 μg/ml recombinant lipocalins or a defined cytokine cocktail consisting of TNFα (10 ng/ml; kindly provided by Dr G. R. Adolf, Bender, Vienna, Austria), IL-1β (2 ng/ml; PeproTech EC Ltd, London, UK), IL-6 (1000 U/ml; PeproTech) and PGE2 (1 μg/ml, Prostaglandin E2; Pharmacia & Upjohn SA, Buurs, Belgium) as maturation stimulus. Preparation of T cells: Bulk T cells were isolated from the rosettes that had formed with neuraminidase-treated sheep red blood cells during the monocyte isolation procedure by lysing the sheep red blood cells with ammonium chloride as described 21. Naïve CD4^+^ T cells were isolated from bulk T cells using a panning technique described previously 22,23.

Co-cultures of untreated or lipocalin-treated (10 μg/ml, 48 hrs) human monocyte-derived DCs and allogeneic naïve T cells were performed in flat bottom 24 well plates using 2.5 × 10^5^ DCs and 1 × 10^6^ T cells. Supernatants and cells are harvested after 5 days for further analyses.

### Immunocytochemical analysis, confocal microscopy

Live-cell imaging: DCs were seeded into a chambered cover glass slide (Thermo Fisher Scientific). Fluorescent dye (Alexa 488 and/or Alexa 594) labelled recombinant lipocalins (10 μg/ml) and organelle stains (lysotracker red or transferrin red, Invitrogen) according to the manufacturer’s protocol, were added for 1 hr.

Fixed cell imaging: DCs were allowed to adhere to poly L-Lysine (Sigma-Aldrich, St. Louis, MO, USA; 100 μg/ml for 1 hr at 37°C) coated cover glass slides within a 24-well-plate for 30 min. Fluorescent dye (Alexa 488 and/or Alexa 594) labelled recombinant lipocalins (10 μg/ml) were added for 1 hr together with unlabelled anti-major histocompatibility complex class II (MHC II) antibody to saturate surface MHC class II binding sites. Cells were fixed in Fix&Perm solution (An Der Grub Bio research GmbH, Kaumberg, Austria) containing 1 μg/ml 4′,6-Diamidin-2-phnylindol (DAPI) for 10 min. followed by immunostaining performed with a Fluorescein isothiocyanate (FITC) labelled anti-human-MHC II antibody to stain for intracellular MHC class II molecules.

Confocal Microscopy was performed with a spinning disk confocal system (UltraVIEW VoX; Perkin Elmer, Waltham, MA, USA) connected to a Zeiss AxioObserver Z1 microscope (Zeiss, Oberkochen, Germany). Images were acquired with the Volocity software (Perkin Elmer) using a 63× oil immersion objective with a numerical aperture of 1.4.

### FACS analyses

Fluorescence activated cell sorting (FACS) analysis of surface molecules were performed with directly labelled primary antibodies specific for CD40, CD80, CD86 and MHCII (BD Pharmingen, San Diego, CA, USA) CD83 (Immunotech/Coulter, Fullerton, CA, USA) on untreated or lipocalin-treated (10 μg/ml, 48 hrs) DC. Specimens were analysed on a FACS Canto II and data handled with FlowJo software (BD Immunocytometry Systems, San Jose, CA, USA).

### Tryptophan breakdown

Tryptophan and kynurenine concentrations in supernatants were determined by reversed-phase high performance liquid chromatography as described earlier 24. Specimens were deproteinized with trichloroacetic acid and were separated on reversed-phase C18 material using 15 mmol/l acetic acid/sodium acetate (pH = 4.0; Merck), as effluent. Tryptophan was monitored by means of its native fluorescence (Varian ProStar 360; Agilent technology, Palo Alto, CA, USA) at 285 nm excitation and 360 nm emission wavelengths. Kynurenine was detected by ultraviolet absorption (Shimadzu SPD-6A, Korneuburg, Austria) at 365 nm wavelength in the same chromatographic run. Finally, as an estimate of IDO (indoleamine 2,3-dioxygenase) activity, kynurenine to tryptophan ratio (Kyn/Trp) was calculated and expressed as μmol kynurenine per mmol tryptophan 25.

### ELISA

Culture supernatants of untreated or lipocalin-treated (10 μg/ml, 48 hrs) human monocyte-derived DCs were analysed for IL-12 (p70). Cell supernatants of co-cultures of human monocyte-derived DCs with allogeneic CD45RA^+^ naïve T-cells (5 days) were analysed for IFN-γ and IL-13. ELISA kits were from BD Pharmingen, San Diego, CA, USA.

### Affymetrix GeneChip analysis

The microarray data set was generated at the Expression Profiling Unit of the Medical University Innsbruck. All experimental steps were executed according to the manufacturer’s protocols. Two hundred nanograms per sample of total RNA isolated from untreated, matured (maturation cocktail 48 hrs) or lipocalin-treated (10 μg/ml, 48 hrs) DCs using TRIzol reagent (Invitrogen) were processed using the Ambion Affymetrix GeneChip WT Expression Kit (Part no. 4411974; Ambion, Life technology, Carlsbad, CA, USA) and hybridized with a total of 4 Affymetrix Human Gene ST 1.0 GeneChips in an Affymetrix hybridization oven. The microarrays were stained in an Affymetrix fluidic station 450 and fluorescence signals recorded in an Affymetrix scanner 3000. Microarray data were pre-processed using the ‘generalgcrma’ package 26 and a custom transcript-level ‘CEL definition file’. Raw and pre-processed microarray data have been deposited at the Gene Expression Omnibus (accession number GSE62436). Moderated statistics based on linear models, implemented in Bioconductor’s limma package 27 were employed to perform differential gene expression analysis. The microarray experiment was performed in biological duplicates based on 2 different pools of DC preparations of three donors each. Can f 1 and Lcn-1 treated samples as described above were obtained from the same donor. To account for variability originating from the differences between the pools rather than from the differences between the two treatment conditions, a categorical variable representing the assignment of samples to the pools was included into the linear regression models. Subsequently the obtained p-values were adjusted for multiple hypotheses testing based on the method of Benjamini and Hochberg 28 to strongly control the false discovery rate. Genes with a raw p-value of smaller than 0.05 and an absolute M-value greater than 1 were considered as potentially interesting.

### mRNA quantification

RNA was isolated using TRIzol reagent (Invitrogen) and cDNA was synthesized with Superscript II (Invitrogen, Vienna, Austria) from total RNA. Detection of formyl peptide receptor (FPR)3 was performed with the following oligonucleotides chosen with the help of the primer search tool Primer3 (http://primer3.ut.ee/): Fwd: 5′ TGGTGTGGGAAGATGGAAACC and Rev: 5′ CAGATGGTGTTGACTGTGCG.

Real-time PCR analyses to quantify the expression of mRNAs were performed in triplicates on a CFX96 real-time system using the SsoAdvanced SYBR® Green Supermix Kit from BioRad (Bio-Rad, Hercules, CA, USA). After normalization of the data according to the expression of Glyceraldehyde 3-phosphate dehydrogenase (GAPDH) mRNA, the relative expression levels of mRNAs were calculated.

### Statistics

Data were handled with the PRISM software (GraphPad software, San Diego, CA, USA) and paired student’s *t*-tests were performed to evaluate significance.

## Results

### Uptake and cellular targeting of lipocalins in human monocyte-derived DCs

**Figure 1:**
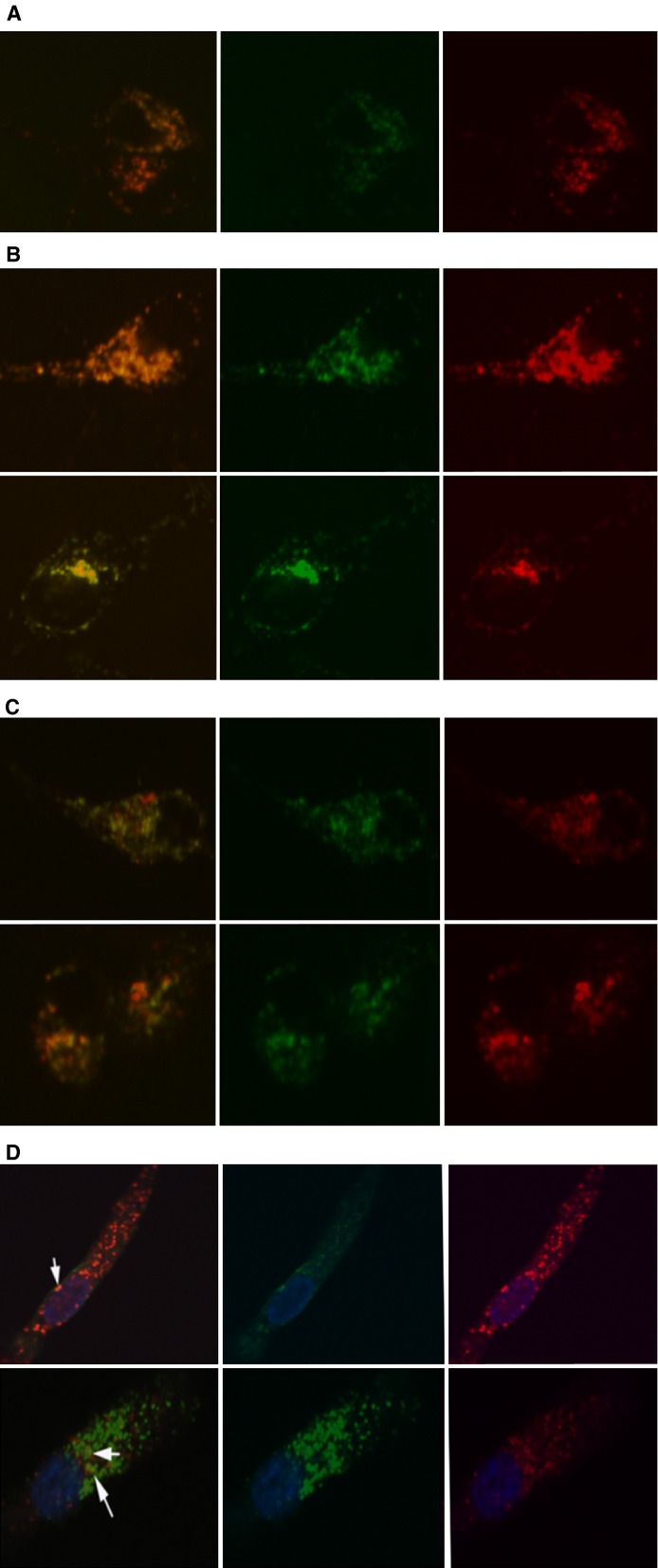
Intracellular localization of lipocalins in dendritic cell (DC). Lipocalins and cell organelle trackers were added to day 6 immature DCs for 1 hr in chambered cover glass slides. Live cells (A–C) were analysed for internalized fluorescence. Immobilized cells (D) were incubated with labelled lipocalins for 1 hr, permeabilized and stained for MHC classII. (A) Uptake of Lcn-1 and Can f 1 by immature DCs. Lcn-1 labelled with Alexa 488 (green) and Can f 1 labelled with Alexa 594 (red). Left panel: overlap, middle panel: Lcn-1, right panel: Can f 1. (B) Lcn-1 and Can f 1 co-localization with transferrin. Alexa 488-Lcn-1 (green, upper panel) or Alexa 488-Can f 1 (green, lower panel) were added to DCs together with Alexa 647-tranferrin (red). Left panel: overlap, middle panel: Lcn-1 or Can f 1 (green), right panel: transferrin (red). (C) Lcn-1 and Can f 1 co-localization with lysotracker. Alexa 488-Lcn-1 (green, upper panel) or Alexa 488-Can f 1 (green, lower panel) were added to DCs together with lysotracker red (red). Left panel: overlap, middle panel: Lcn-1 or Can f 1 (green), right panel: lysotracker (red). (D) Localization of lipocalins and intracellular MHC class II staining. Alexa 594 labelled Lcn-1 (red, upper panel) or Alexa 594-Can f 1 (red, lower panel) containing vesicles rarely also stain for MHC class II (green, see white arrows for double positive vesicles).

To evaluate whether human monocyte-derived DCs are able to take up recombinant lipocalins, they were labelled with fluorescent dyes and immature DCs were incubated with the lipocalins in chambered coverglass slips for 1 hr. Confocal imaging from live DC preparations shows internalization of the fluorescence labelled lipocalins Lcn-1 and Can f 1 into the same vesicles (Fig. 1A). Furthermore, co-localisation studies with transferrin as a marker for the endosomal pathway (Fig. 1B) and lysotracker for lysosomes (Fig. 1C) as well as with MHC class II for the antigen processing and presentation pathway (Fig. 1D) were performed to track the fate of the lipocalins. No differences were observed between Lcn-1 and Can f 1. Complete overlap of both lipocalins (A, Lcn-1 green, Can f 1 red) and of both lipocalins with transferrin (B, Lcn-1 green transferrin red and Can f1 green, transferrin red) as well as partial overlap with lysotracker (C, Lcn-1 green lysotracker red and Can f 1 green lysotracker red) could be observed by live-cell confocal imaging. Confocal imaging of fixed cells shows occasional co-localization of both lipocalins with MHC class II molecules (D, Lcn-1 red MHC class II green and Can f 1 red MHC class II green). Temperature dependent active (37°C *versus* 4°C as a negative control) internalization is shown in Figure S2.

### Lipocalins induce expression of several maturation markers in human monocyte-derived DCs but to a different extent

To evaluate the consequence of lipocalin uptake the maturation status of DCs and the capacity of DCs to induce T-cell proliferation were measured.

**Figure 2:**
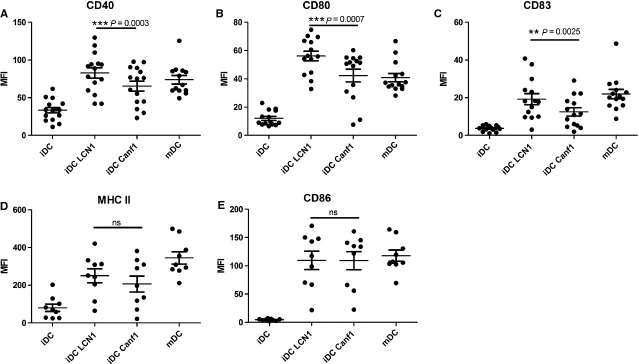
Lcn-1 induced maturation marker expression on DCs is different from Can f 1 induced expression. Median of fluorescent intensity (MFI) of FACS analyses for the indicated maturation markers are shown for untreated or lipocalin-treated (10 μg/ml, 48 hrs) DCs each dot representing a single donor. DCs matured with pro-inflammatory cytokines and PGE2 (maturation cocktail) were used as a positive control. Can f 1 induces significantly less expression of CD40 (A), CD80 (B) and CD83 (C) in DCs than Lcn-1, while MHC class II (D) and CD86 (E) expression is similar. iDC: immature DCs; mDC: DCs treated with maturation cocktail. *P < 0.05; **P < 0.01; ***P < 0,001

Immature DCs were treated with 10 μg/ml recombinant lipocalins for 48 hrs and the expression of maturation markers was measured. Both lipocalins induced expression of the most prominent maturation markers as indicated (Fig. 2). The magnitude of the induction for CD40, CD80 and CD83, however, is significantly higher in DCs treated with Lcn-1 than in DCs treated with Can f 1. The magnitude of induction for HLA-DR (MHC II) and CD86, instead, is similar. T-cell proliferation of DC-T cell co-cultures was measured by CFSE dilution assay and was not significantly different in T cells stimulated by DCs treated with Lcn-1 or Can f 1 (data not shown). We also tried to reproduce these results with a commercially available Lcn-1 produced in human cells. However, this was not possible probably because of the fact that these protein preparations seem to contain the entire Lcn-1 including the signal peptide. We hypothesize that this protein differs from mature Lcn-1 in cellular binding or in cellular uptake.

### The allergenic lipocalin Can f 1 induces less tryptophan breakdown and lower production of IL-12 in DCs than Lcn-1

**Figure 3:**
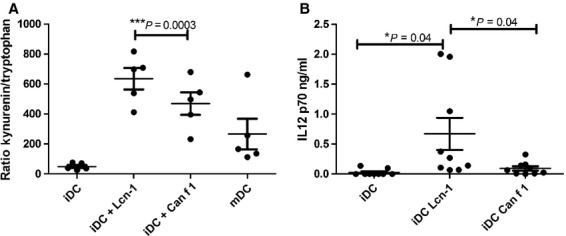
(A) Tryptophan breakdown (kynurenine to tryptophan ratio) is higher in DCs treated with Lcn-1 than in DCs treated with Can f 1. Supernatants of DC cultures treated with Lcn-1 or Can f 1 for 48 hrs were analysed for kynurenine and tryptophan content. The kynurenine to tryptophan ratio is shown. (B) DCs treated with Can f 1 produce significantly less IL12p70 than DCs treated with Lcn-1. Supernatants of DCs treated with Lcn-1 or Can f 1 were subjected to ELISA analyses for IL12p70. DCs treated with the maturation cocktail were used as control. iDC: immature DCs; mDC: DCs treated with maturation cocktail. *P < 0.05; **P < 0.01; ***P < 0,001

The lower expression of maturation markers induced on DCs treated with the allergenic Can f 1 as compared to those treated with endogenous Lcn-1 lead us to further investigate the conditions provided by DCs for T-cell stimulation. One indicator for T-helper cell subtype induction is the IDO pathway for tryptophan breakdown 29. Tryptophan and kynurenine concentrations in supernatants of human monocyte-derived DCs were measured and found to be significantly lower in DCs treated with Can f 1 as compared to DCs treated with Lcn-1 (Fig. 3A).

Another indicator for the quality of the immune response induced are the cytokines released by DCs. The bioactive IL-12p70 is a prerequisite for Th1 induction 2. Supernatants of DCs treated with Lcn-1 or Can f 1 were evaluated for their IL-12 content. Significantly less IL-12 was produced by DCs treated with Can f 1 than by DCs treated with Lcn-1 (Fig. 3B).

### IFNγ and IL-13 production of Th cells are differentially induced by DCs treated with Lcn-1 or Can f 1

**Figure 4:**
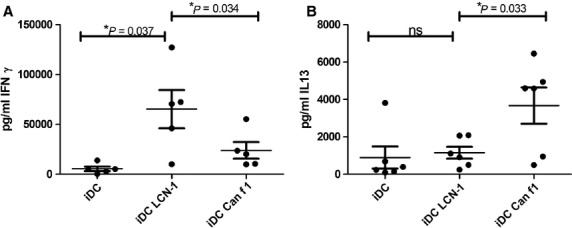
CD4 positive naïve T cells develop into Th1 cells when primed by DCs activated with Lcn-1 and into Th2 T cells when primed with DC activated with Can f 1. Supernatants of DC-T cell co-cultures of the same 5 individual donors were analysed by ELISA for IFNγ (A) and IL13 (B) concentrations. iDC: immature DCs. *P < 0.05; **P < 0.01; ***P < 0,001

Allogeneic co-cultures were chosen to elicit immune responses against the human Lcn-1 to be compared with immune responses induced by Can f 1. To analyse the outcome of the immune response, the key cytokines IFNγ for Th1 and IL13 for Th2 cells were measured in supernatants of DC-T cell co-cultures (Fig. 4). CD4 positive naïve T cells differentiated into Th1 cells when activated by DCs treated with the non-allergenic Lcn-1 and into Th2 cells when activated with the allergenic Can f 1. No significant differences were found for the induction of regulatory T cells measured by CD4, CD25 and FoxP3 expression in FACS analysis and in percent demethylated FoxP3 exon 1 encoding DNA (data not shown).

### Confirmation of differential effects of lipocalins on DCs with Fel d 4 and human MUP

**Figure 5:**
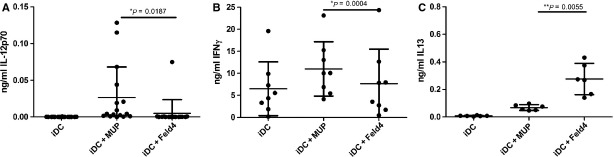
Verification of divergent effects of allergenic and non-allergenic lipocalins on DC: IL12 (A) production and TH subset induction shown by IFNγ (B) and IL13 (C) production of T cells stimulated with the homologous lipocalins Fel d 4 and MUP. *P < 0.05; **P < 0.01; ***P < 0,001

The same conditions as for Lcn-1 and Can f 1 were applied for further studies with another pair of homologous lipocalins, the cat dander lipocalin Fel d 4 and a putative human homologue named MUP, sharing more than 60% sequence homology. These lipocalins exert activation pattern on human monocyte-derived DCs similar to those of Can f 1 and Lcn-1: a TH2 prone activation of DCs with the allergen Fel d 4 and a TH1 prone activation with its putative human homologue MUP with regard to IL-12 production and TH subset induction (Fig. 5).

### Global gene expression analyses confirm functional data and reveal significant differences in the expression of components of the trafficking, sorting and antigen presentation machinery

Among the molecules found to be up-regulated in Lcn-1 treated DCs as compared to Can f 1 treated DCs we found IL-12 p35, IDO2 (see raw and pre-processed microarray data deposited at the GEO, accession number GSE62436) and others, that support and help explain our functional data described above. The pathway analyses performed on the more than 24,000 gene expression data obtained by the microarray experiment strongly indicate differences in intracellular trafficking, sorting and antigen presentation (Table 1). The gene encoding FPR3, accession number NM_002030.3, was one of the genes highly expressed in Can f 1 stimulated DC compared to Lcn-1 treated DC. This was verified by real-time PCR (Fig. S3).

**Table 1:** Pathway analyses sorted by *P*-value and average (avg) L-fold change

ReactomeID	AvgLFC	Strength	*P*-value	Reactome name
1236973	2.96076	7.833437	0.013728	‘Homo sapiens: Cross-presentation of particulate exogenous antigens (phagosomes)’
‘444473’	1.171457	2.86947	0.034643	‘Homo sapiens: Formyl peptide receptors bind formyl peptides and many other ligands’
‘196819’	1.02666	2.29568	0.047826	‘Homo sapiens: Vitamin B1 (thiamin) metabolism’
‘177504’	0.95006	3.29113	0.046679	‘Homo sapiens: Retrograde neurotrophin signalling’
‘217378’	0.890100	5.34060	0.00890	‘Homo sapiens: Binding and Uptake of Ligands by Scavenger Receptors’
‘77346’	0.889308	1.98855	0.03370	‘Homo sapiens: Beta oxidation of decanoyl-CoA to octanoyl-CoA-CoA’

## Discussion

Induction of allergic responses characterized by the development of the Th2 helper cell subtype is not yet sufficiently understood. Data suggest the influence of individual susceptibility to allergens and the circumstances at the time-point of T-cell activation as well as the crosstalk between DCs and allergens to be critical for the decision to drive a specific T-helper cell subtype 30–32. In this study we accumulate evidence that the antigen itself is crucial for the modulation of DCs to induce T-helper cell differentiation. We compared the effects of Lcn-1 and Can f 1, two homologous lipocalins 13 with different allergenic potential, on human monocyte-derived DCs.

No significant differences in subcellular localization of the lipocalins were found (Fig. 1). Both lipocalins co-localize almost entirely with transferrin in endosomes. Partial co-localization with lysotracker indicates fusion of some of the lipocalin containing endosomes with lysosomes. Few vesicles containing lipocalins and MHC class II molecules show that a part of the lipocalins enters the presentation machinery of DCs 33,34. Uptake of lipocalins induced maturation marker expression such as CD86 and MHC II to a similar extent. However, non-allergenic Lcn-1 triggered changes in DCs which can be interpreted as Th1 inducing, while the allergenic Can f 1 initiated effects which can be attributed towards a Th2 immune response. These changes include expressions of the maturation markers CD40, CD80 and CD83, tryptophan breakdown and production of the cytokine IL-12.

In detail, the co-stimulatory molecule-expression of CD40, CD80 and CD83 was significantly lower in DCs stimulated with Can f 1 (Fig. 2). These cells are thus quantitatively less mature than DCs treated with Lcn-1. This quantitative difference in maturation of DCs has been shown to dictate Th2 polarization 35. As shown in Figure 3A tryptophan breakdown was significantly higher when measured in supernatants of DC cultures incubated with Lcn-1 than in supernatants of Can f 1 treated DCs. This additional indicator of stronger DC maturation 36 and its close relation to IFN-γ production 37 are indicative for Th1 polarization. Similar results were obtained measuring the levels of IL-12 concentrations in supernatant of DCs treated with either Lcn-1 or Can f 1 (Fig. 3B). We found significantly elevated levels of IL-12 in the supernatant of Lcn-1 treated DCs indicative for a Th1 differentiation. The results obtained for DC-lipocalin crosstalk regarding maturation, tryptophan breakdown and IL-12 production are consistent and indicate that T-helper cell differentiation by DCs treated with the endogenous Lcn-1 is prone for the Th1 subtype, while T-helper cell differentiation by Can f 1 treated DCs is directed towards the Th2 subtype.

To verify the distinct Th cell inducing capacities the supernatants of allogeneic co-cultures of naïve CD4^+^ CD45RA^+^ T cells stimulated with Lcn-1 or Can f 1 treated DCs were analysed for IFNγ as Th1 cytokine and IL-13 as Th2 cytokine (Fig. 4). As expected, supernatants of co-cultures with Lcn-1 treated DCs contained significantly more IFNγ but less IL-13 than the co-cultures of naïve T cells and Can f 1 treated DCs. Taken together our data show that the effect of the lipocalins on DCs alone has the power to direct T cell polarization.

The effects of lipocalins on DCs have been discussed differentially in recent reports. Although most of the data shown in these reports are not directly comparable to our study, some are still contradictory. Virtanen and colleagues found that mammalian lipocalin allergens do not exhibit DC-activating capacity and found no differences in the CD4^+^ T cell responses to the two homologous lipocalins 38,39 while Preston *et al*. 40 found immunomodulatory activity of hard tick-derived lipocalins and Roth-Walter *et al*. found that the lipocalin cow allergen holo-Bos d 5 and the lipocalin-like holo-Bet v2 promote Th2 cells 41,42. In our study we confirm DC-modulatory activity by a second pair of homologous lipocalins, the cat dander allergen Fel d 4 and its putative human homologue MUP. These homologous lipocalins showed similar results like Lcn-1 and Can f 1 regarding the induction of IL-12 and T-helper cell subtypes (Fig. 5) and Liukko *et al*. 38 show that lipocalin derived peptides alone are not able to drive differential immune responses which supports our view that the interaction of lipocalins with DCs is essential to define the type of immune response. More studies might be necessary to clear these discrepancies. Given the functional plasticity of DC 43,44 continuing research might reveal the significance of the differences seen between allergenic and non-allergenic lipocalins. As a first step a broader insight in the changes induced in DCs exposed to allergenic or non-allergenic lipocalins was gained by our gene expression data analyses. An overall cellular function of the genes combining the significantly different reactomes (Table 1) is their relevance in endocytosis. Over the past decade a major role for the endosomal system has emerged for the regulation of innate and adaptive immune systems. Antigen-presenting cells take up antigen *via* receptor mediated endocytosis or fluid phase macropinocytosis into endosomes where it is partially degraded, endosomes fuse with lysosomes and the antigen is finally loaded onto MHC II molecules for delivery to the cell surface for T-helper cell activation. During this process endosomal signalling is relevant for the initiation of both, innate and adaptive immunity to regulate cargo sorting and trafficking as well as communication of endosomal compartments with signalling networks 45,46. Therefore, differences in the expression profile of molecules involved in endocytosis and endosomal maturation may well reflect differential processing of antigen and induction of immune responses 47.

Interestingly, the gene encoding FPR3, accession number NM_002030.3, was one of the genes highly expressed in Can f 1 stimulated DC compared to Lcn-1 treated DC. This protein is a member of the human N-formyl peptide receptors, which belong to the G-protein-coupled chemo-attractant membrane receptors. These receptors have been found to be involved both in infective and inflammatory processes by the binding of microbial peptides and other ligands. However, recent investigations could not detect G-protein activation in FPR3 expressing cells and found that it is, in contrast to FPR1 and 2, constitutively internalized. It was therefore suggested that FPR3 may serve as an alternate, ligand scavenging function similar to some chemokine receptors 48,49. Our data from the expression analysis (Fig. S1) together with a clear punctual intracellular staining and co-localization of FPR3 with lipocalins (data not shown) suggest a role of FPR3 in the targeting/processing of lipocalins in DC and will be a subject of further studies.

## Supporting Information

**Figure S1.** Size estimate for recombinant lipocalins.

**Figure S2.** Active uptake of lipocalins by DC.

**Figure S3.** FPR3 mRNA expression in DC in response to lipocalins.
