# Supplementary material for: Allergenic Can f 1 and its human homologue Lcn-1 direct dendritic cells to induce divergent immune responses
Source: J Cell Mol Med. 2015 Jul 27;19(10):2375–84. doi: 10.1111/jcmm.12616 (PMC4594679; doi:10.1111/jcmm.12616)
Supplement: Supplementary file 4 [file jcmm0019-2375-sd4.docx]

Legends to

Supplemental figure 1: Estimated size of recombinant lipocalins

Coomassie blue stained SDS PAGE of 20 µg lipocalins per lane: Lane 1: Can f 1, Lane 2: Fel d 4, Lane 3: Fel d 4-like, Lane 4: Lcn1

Supplemental figure 2: Active uptake of recombinant lipocalins by dendritic cells

FACS analyses for dendritic cells incubated for 1 h with Alexa 488 labelled lipocalins, left: Can f 1, right: Lcn-1. Dotted line: dendritic cells without lipocalins, dashed line: dendritic cells incubated with Alexa 488 labelled lipocalins at 4°C, solid line: dendritic cells incubated with Alexa 488 labelled lipocalins at 37°C.

Supplemental figure 3: FPR3 mRNA quantification in response to lipocalin treatment

mRNA from monocyte derived DC of 7 different donors treated or not with recombinant lipocalins were reverse transcribed and submitted to FPR3 specific real time PCR. Each point per column in the graph represents an individual donor
